# Imported Chikungunya Infection, Italy

**DOI:** 10.3201/eid1308.070161

**Published:** 2007-08

**Authors:** Anna Beltrame, Andrea Angheben, Zeno Bisoffi, Geraldo Monteiro, Stefania Marocco, Guido Calleri, Filippo Lipani, Federico Gobbi, Francesca Canta, Francesco Castelli, Maurizio Gulletta, Sara Bigoni, Veronica Del Punta, Tiziana Iacovazzi, Roberto Romi, Loredana Nicoletti, Maria Grazia Ciufolini, Giada Rorato, Camilla Negri, Pierluigi Viale

**Affiliations:** * Clinic of Infectious Diseases at University of Udine, Udine, Italy; † Sacro Cuore Hospital, Negrar, Italy; ‡ Amedeo di Savoia Hospital, Torino, Italy; § University of Brescia, Brescia, Italy; ¶ F. Fallacara Hospital, Triggiano, Italy; # Istituto Superiore di Sanità, Roma, Italy

**Keywords:** Chikungunya, travelers, Italy, letter

**To the Editor:** Chikungunya virus (CHIKV) infection is a self-limiting illness characterized by fever, headache, weakness, rash, and arthralgia. Some patients have prolonged weakness or arthralgia lasting several months. In 2006, several Indian Ocean states and India had an outbreak of CHIKV infection (*1*,*2*). During the epidemic’s peak, some European and American travelers returning from these areas were infected (*3*–*6*).

Because the foci of *Aedes albopictus,* 1 of the 2 main vectors of CHIKV, are now in Italy and many travelers visit CHIKV-epidemic areas, surveillance for imported cases is mandatory in Italy (*7*). From July to September 2006, a total of 17 confirmed cases of CHIKV infection were observed in travelers at 5 Gruppo di Interesse e Studio delle Patologie di Importazione (GISPI) centers (Italian network of Institutes of Infectious and Tropical Diseases). Serologic diagnosis was performed with a hemagglutination-inhibition test and confirmed by a plaque-reduction neutralization test (*8*). Demographic and epidemiologic characteristics of these patients are reported in the Table.

**Table:** Demographic and epidemiologic data on 17 travelers with chikungunya infection diagnosed in 2006, Italy

Patient no.	Sex	Age, y	Reason for travel	Country of origin	Date of return (length of stay, d)	Date of first medical assessment after return (delay, d)	Last date of fever (length of fever, d)	Fever on date of return?
1*	M	32	Business	Réunion	Feb 23 (93)	Feb 25 (2)	Feb 26 (4)	Yes
2†	F	39	Tourism	Mauritius	Feb 28 (10)	Feb 28 (0)	Feb 28 (4)	Yes
3‡	M	46	Tourism	Mauritius	Mar 7 (10)	Mar 7 (0)	Mar 6 (5)	No
4‡	M	32	Tourism	Madagascar	Mar 7 (15)	Mar 8 (1)	Mar 4 (4)	No
5§	M	49	Tourism	Mauritius	Mar 08 (16)	Mar 15 (7)	Mar 4 (5)	No
6‡	M	66	Tourism	Madagascar	Mar 24 (15)	Mar 24 (0)	Mar 27 (5)	Yes
7§	M	36	Tourism	Mauritius	Apr 4 (15)	Apr 5 (1)	Apr 1 (6)	No
8*	F	43	Resident	Madagascar	Apr 10 (–)	Apr 11 (1)	Mar 2 (6)	No
9‡	F	46	Tourism	Réunion	Jan 30 (16)	Apr 13 (73)	NA (2)	–
10¶	F	44	Visit relatives	Mauritius	Apr 17 (33)	Apr 19 (2)	Apr 7 (12)	No
11‡	F	36	Tourism	Mauritius	Apr 30 (11)	May 3 (3)	May 3 (3)	Yes
12‡	M	31	Tourism	Réunion	Apr 21 (30)	May 4 (13)	Apr 5 (6)	No
13‡	M	44	Visit relatives	Cameroon	May 3 (24)	May 22 (19)	May 7 (6)	Yes
14*	M	35	Tourism	Seychelles	May 31 (9)	Jun 1 (1)	Jun 1 (2)	Yes
15*	M	38	Tourism	Mauritius	May 10 (11)	Jun 12 (2)	May 7 (4)	No
16‡	F	58	Missionary work	Central African Republic	Jun 24 (14 y)	Jul 10 (16)	Apr 26 (12)	No
17*	F	57	Business	India	Sep 8 (31)	Sep 9 (1)	Sep 10 (4)	Yes

*GISPI (Gruppo di Interesse e Studio delle Patologie di Importazione) centers: Torino. †GISPI center: Udine. ‡GISPI center: Negrar. NA, not available. §GISPI center: Brescia. ¶GISPI center: Triggiano.

Cases were distributed throughout the year with a peak from March to May 2006 (n = 10). Nine patients (53%) were men. Median age was 43 years (range 31–66 years). Several reasons for travel were reported: tourism (64.6%), visits to relatives or friends (11.8%), business (11.8%), and missionary work (5.9%). One patient was a resident in the disease-epidemic area. The median exposure time in the CHIKV-endemic area for the 15 travelers was 15 days (range 9–93 days) (missionary and resident patients were excluded). The median delay before being seen at a clinic after return was 2 days (range 0–73 days). Only 7 patients (41.2%) were hospitalized. The remainder were outpatients.

All patients had fever; arthralgia (88.2%, n = 15), weakness (70.6%, n = 12), headache (11.8%, n = 2), diarrhea (11.8%, n = 2), and gum bleeding and epistaxis (5.9%, n = 1) were other reported symptoms. The median duration of fever was 5 days (range 2–12 days). Only 7 of 16 patients (43.8%) were still febrile when first seen. Physical examination showed diffuse macular erythematous rash in 13 patients (76.5%), a similar rate to that reported among French travelers (*4*). Hepatomegaly was found in 2 patients (11.8%), splenomegaly in 2 (11.8%), and peripheral lymphadenopathy in 2 (11.8%).

Twelve acute-phase patients were admitted to the hospital for blood testing within 3 days of the initial examination. In contrast with results of other studies, leukopenia and thrombocytopenia were uncommon in our study. Leukopenia (leukocyte count <4,000/μL) was present in 4 patients (33.3%) and thrombocytopenia (platelet count <150,000/μL) in 1 patient (8.3%). This finding may help distinguish CHIKV infection from dengue fever (*4*). Anemia (hemoglobin level <12 g/dL) was found in only 1 patient (8.3%). Alanine aminotransferase (ALT) and aspartate aminotransferase (AST) determination were available for 12 patients. ALT and AST levels were elevated (>40 IU/L) in 5 (41.7%) and 2 (16.7%) patients, respectively. Seven (46.7%) of 15 patients fully recovered within 1 month; 8 patients (53.3%) reported persistent arthralgia.

Because the GISPI network provides regional coverage only, the number of imported CHIKV cases in all of Italy in 2006 was likely higher. Moreover, most patients probably did not seek medical care, and when they did, physicians may have failed to recognize the disease because of lack of familiarity with it or limited diagnostic facilities. Differential diagnosis with other arthropodborne viruses of the *Alphavirus* genus (Ross River, Barmah Forest, o’nyong nyong, Sindbis, and Mayaro viruses) is difficult, but these are comparatively rare. In contrast, dengue and CHIKV epidemics may overlap, and potential patients should be screened for both.

The potential risk for introduction and establishment of CHIKV reservoirs in areas with mosquito vectors was discussed in March 2006 by a multidisciplinary European expert panel (*9*). In Italy, *A. albopictus* was first recorded in 1990; it has since quickly spread across the country. Scattered foci are now reported in almost all regions, mainly along the coastal plains, from the sea to the inlands, up to an altitude of ≈500–600 m (*7*).

The ability of *A. albopictus* to colonize new areas and its adaptability to the mild Italian climate allow vector populations to be active throughout the year (*10*). The patient is thought to be viremic for only 6–7 days (shortly before and during the febrile period) (*6*). We were unable to directly assess viremia levels; however, almost half the patients were still febrile on return to Italy, which suggests a potential risk.

Although the same mosquito is a potential vector of dengue, no autochthonous case has been reported as yet, despite annual reports of many imported dengue cases in Italy. On the other hand, the clinical manifestations of both conditions are nonspecific, and a hypothetical autochthonous case would most likely go undiagnosed unless a targeted surveillance system were established. Prompt reporting of imported CHIKV infections is essential for monitoring of potential risk. The possibility of introducing CHIKV into Italy cannot be ruled out on the basis of current evidence.
